# Diet and Oxidative Status. The Dietary Pattern and Urinary 8-Isoprostane in Healthy Spanish Women

**DOI:** 10.3390/antiox8080271

**Published:** 2019-08-02

**Authors:** Nuria Ruiz, Ana Belén Segarra, Luis Lara, Manuel Ramírez-Sánchez, Isabel Prieto

**Affiliations:** 1 Neuroendocrinology and Nutrition Research Group. Department of Health Science, University of Jaén, 23071 Jaén, Spain; 2 Department of Physiology and Biochemistry of the Animal Nutrition, Estación Experimental del Zaidín. Spanish Council for Scientific Research (CSIC), Armilla, 18100 Granada, Spain

**Keywords:** Mediterranean diet, extra virgin olive oil, urine 8-isoprostanes

## Abstract

The Mediterranean diet is associated with a low incidence of physiologic and metabolic non-communicable diseases such as hypertension, obesity, and insulin resistance. These chronic diseases are closely related to oxidative status, which is determined by the balance between oxidant and antioxidant levels. The Mediterranean diet is rich in foods with important antioxidant properties, such as fruits and extra virgin olive oil. The aim of this work was to establish the relationship between dietary patterns, the total intake of polyphenols, and the levels of 8-isoprostanes in urine, as a marker of lipid peroxidation, in a group of healthy Spanish women. The main sources of dietary polyphenols were fruits, vegetables, pulses, nuts, and extra virgin olive oil. There was a significant and positive correlation between the estimated intake of polyphenols, total polyphenols excreted in urine, adherence to the Mediterranean diet, and the intake of specific food groups. A positive correlation was established between the total polyphenols in urine and the intake of raw extra virgin olive oil. However, a negative correlation was established between the amount of 8-isoprostanes in urine, total intake of polyphenols, adherence to the Mediterranean diet, and the intake of fruits and nuts. These results indicate an association between oxidative status and the intake of foods that are typical of the Mediterranean diet, in healthy women. Furthermore, the results demonstrate the use of urine 8-isoprostanes as a marker of adherence to the Mediterranean diet.

## 1. Introduction

The Mediterranean diet is characterized by a high intake of plant foods, such as vegetables, fruits, nuts and seeds, cereals, and extra virgin olive oil (EVOO), as the main source of dietary fat. However, a moderate intake of animal foods, such as dairy products, poultry and other meats, eggs, fish and shellfish, is also part of this dietary pattern [1,2]. Plant foods provide an excellent source of antioxidants, which preserve foods from farm to plate and maintain the stability of nutrients, and the functionality of other minor components [3]. More importantly, they provide a protective role against most chronic diseases, such as diabetes and hypertension, which has been demonstrated in several studies [4], and supports the role of the Mediterranean diet as one of the healthiest dietary models available around the world.

Within the antioxidant components of the Mediterranean diet, polyphenols stand out. Usually, polyphenols are divided into several groups, contained in high amounts in fruits [5,6], as well as other dietary sources such as vegetables, legumes, whole grains, and EVOO; all foods typical of the Mediterranean diet [7,8]. The olive tree (*Olea europaea* L., family *Oleaceae*) produces its own battery of polyphenols. These polyphenols are found in EVOO, and include hydroxytyrosol, tyrosol, oleuropein, and oleocanthal [9,10]. The intake of these phenol compounds within the diet has been profusely related to a decrease in chronic and metabolic diseases [3,4,8,11,12], in addition to changes in the gut microbiome, which is also linked to the development of metabolic syndrome [13,14].

These health benefits of polyphenols are related to an increase of total antioxidant status and a decrease in inflammation in the body. High levels of total polyphenol excretion are associated with lower levels of plasmatic triglycerides, glucose, and diastolic blood pressure [15]. Other studies have also shown the relationship between levels of different polyphenols in urine and a lower risk of metabolic diseases [16,17,18].

The main objective of the present study was to analyze, in a group of healthy Spanish women, the relationship between dietary patterns (mainly the daily intake of the typical Mediterranean food groups rich in polyphenols and the total intake of polyphenols) and antioxidant status (determined by the Folin-Ciocalteu assay in urine samples), as well as levels of 8-isoprostanes in urine, as a marker of lipid peroxidation.

## 2. Materials and Methods 

### 2.1. Subjects

This study included 207 adult women volunteers from Jaén (south of Spain), with no previous relevant illnesses, within the age range of 20–60 years. The sample size was calculated according to confidence intervals. The following exclusion criteria were considered: Age under 20 or over 60 years old, abdominal hernia, an increase or decrease of 5 kg or more in bodyweight during the last six months, type 1 diabetics, cardiac insufficiency, hepatic insufficiency or neoplastic disease, treatment with insulin or incretin therapy, and heavy smokers. The participants provided written informed consent and the study protocol was approved by the University of Jaén Ethic Committee and the Andalusian Biomedical Research Ethic Committee (Code number: 1799-N-18). Anthropometric measurements were made according to standardized procedures of the International Standards for Anthropometric Assessment (ISAK, 2001) [19]. Bodyweight was measured using an electronic scale (±0.1 kg). Body Mass Index (BMI) was calculated as weight divided by height squared (kg/m^2^). The criteria used to define overweight were the ones of the World Health Organization (WHO). The population was divided into three generational groups: Group 1, aged 20–30 years (51.5%); Group 2, aged 31–40 years (20.8%); and Group 3, aged 41–60 years (27.7%).

### 2.2. Dietary Assessment

Each participant completed a questionnaire to collect information on diet, lifestyle characteristics, and anthropometric measurements. Less than 9% of the subjects were light smokers (under 10 cigarettes/day). Dietary intake data were collected by face-to-face interviews to estimate usual intake over the previous 3 months. Photographs were included to facilitate the estimation of portion sizes intake. 

The intake of polyphenols was estimated using the Phenol-Explorer database [20]. For each food group (cereals, legumes, raw vegetables, cooked vegetables, fruits, and EVOO raw/cooked), according to the database, the mean of phenol content was calculated according to the standard portion sizes registered in the Food Frequency Questionnaire (FFQ) and converted into 24-h intake. 

Total polyphenol intake was calculated as the sum of all individual polyphenol intakes from all food sources reported by the FFQ Polyphenol. The polyphenol intakes were expressed in energy-adjusted terms (mg per 1000 kcal/day of total energy consumed).

Participants also filled in a 14-point score questionnaire on adherence to the traditional Mediterranean diet (AMDI) [21]. 

### 2.3. Excretion of Total Phenols in Urine

The first urine sample was collected from the volunteers in the morning using sterilized bottles. The 10 mL urine samples were centrifuged for 5 min (1000 g, at 4 °C). After removal of the sediment, they were fractionated into aliquots and frozen at −80 °C. The urine samples and gallic acid standards (GAE) were diluted with 1mL of water Milli-Q and acidified with 34 μL of hydrochloric acid at 35%. They were then used to load the Oasis^®^ MAX SPE 30 mg cartridges (Waters, Milford, CT, USA). The extraction procedure described for Oasis^®^ MAX cartridges was applied, and 15 μL of the eluted fractions were mixed with 170 μL of Milli-Q water in the thermos microtiter 96-well plate, adding 12 μL of Folin-Ciocalteu (FC) reagent, and 30 μL of sodium carbonate (200 gL^−1^). The mixtures were incubated for 1 h at room temperature in the dark. After the reaction period, 73 μL of Milli-Q water was added with the multichannel pipette. Absorbance was measured at 765 nm in Ultraviolet/Visible (UV/VIS) Optima Fluostar spectrophotometer (BMG LabTech, Ortenberg, Germany) [15,22]. 

### 2.4. Determination of Urine 8-Isoprostanes 

Concentrations of 8-isoprostane were measured in triplicate with a 8-isoprostane enzyme immunoassay kit (Cayman Chemical, Ann Arbor, GM, USA). performed manually, and read through with FluoStar Optima Spectrophotometry. Concentrations were expressed as pg isoprostanes/mg creatinine. For creatinine in urine samples, Jaffé alkaline picrate method was used with a commercial kit (Spinreact, Barcelona, Spain). In the absence of disease, creatinine concentrations in urine are usually very stable, and can be used to estimate the urinary excretion of substances with only spot urine samples.

### 2.5. Statistical Analysis

Data were organized and analyzed using Microsoft Excel and the statistical program IBM SPSS statistic 24 (IBM, Armonk, Nueva York, USA). Data were expressed as the mean ± SEM and represented the contribution percentage of the food groups to the total content of the polyphenols in the diet.

Differences between the three age groups at baseline were tested by Analysis of Variance (ANOVA) and post hoc analysis, with the Fisher’s least significant difference (LSD) test for multiple comparisons. Linear regression analysis was used to assess the relationship between 8-isoprostane concentrations, Gallic Acid Equivalents (GAE) in urine, and total polyphenol intake. Significance was defined as a value of *p* < 0.05.

Finally, in order to study the relationships between the different variables that were measured, and to reduce their number to a few factors that help us to interpret their similarities, a Factorial Analysis (FA) was applied. The method used to extract the factors was the Principal Component Analysis. The rotation method applied was the Varimax rotation. For this statistical analysis, the Stat Graphics Centurion XVI software (Stat Point Technologies, Inc., Dallas, TX, USA, 2013) was used.

## 3. Results

### 3.1. Study of Dietary Patterns 

The mean consumption of plant foods were: cereals (including bread, pasta and rice) 1.9 ± 0.07 portions/day, fruits 1.7 ± 0.01 portions/day, vegetables 1.4 ± 0.04 portions/day, pulses 0.3 ± 0.01 portions/day, and nuts 0.4 ± 0.03 portions/day. The intakes of animal products were: eggs 0.4 ± 0.02 portions/day, meats 0.7 ± 0.03 portions/day, dairy 1.8 ± 0.07 portions/day, and fish and seafoods 0.4 ± 0.01 portions/day. The mean daily intake of EVOO was 5 portions/day (2 ± 0.09 portions raw and 3 ± 0.1 portions cooked). There were individual differences between energy intakes, as well as energy requirements, with a mean energy intake of 1859 kcal/day. However, no relationships were found between energy intake and age, or energy intake and bodyweight.

### 3.2. Total Dietary Polyphenol Intake and Adherence to the Mediterranean Diet

**Figure 1:**
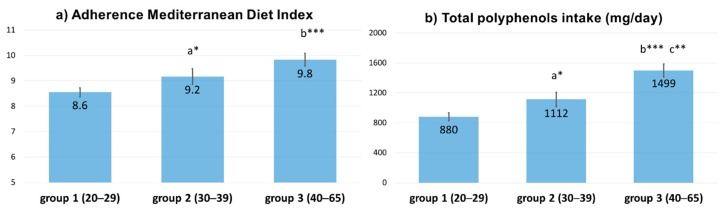
Adherence to the Mediterranean Diet Index (AMDI). (**a**) calculated on a scale 0–14, and total polyphenols intake (**b**), expressed as mg/day for the three age groups considered. a: Difference between group 1 and 2, b: Difference between group 3 and 1, c: Difference between group 2 and 3. * *p* < 0.05, ** *p* < 0.01, *** *p* < 0.001.

Total daily polyphenol intake and the AMDI are shown in Figure 1 for the three age groups considered. The total number of participants available for the final analyses was 207. According to the Phenol-Explorer database, nine typical Mediterranean foods, that contain relevant polyphenols, were included in the questionnaire: Cereals, legumes, potatoes, nuts, raw vegetables, cooked vegetables, fruits, and raw/cooked EVOO. The mean intake of polyphenols was 1098 ± 45 mg/day.

The total polyphenol intake was different between age groups (880 ± 51; 1112 ± 93 and 1499 ± 88 mg/d, groups 1, 2, and 3, respectively; *p* = 0.000), and the AMDI (8.6 ± 0.2; 9.2 ± 0.3 and 9.8 ± 0.2; *p* = 0.000) was higher in the group of older women, based to their higher intake of fruits and vegetables.

**Figure 2:**
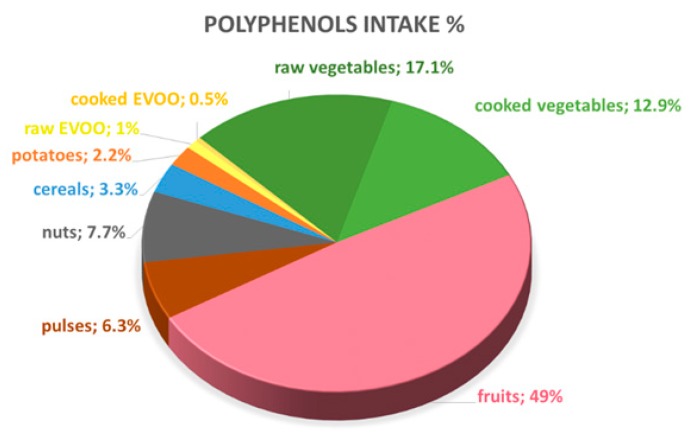
Dietary polyphenol intake corresponded to each Mediterranean food group, expressed as % of total daily polyphenol intake. EVOO = Extra Virgin Olive Oil.

**Figure 3:**
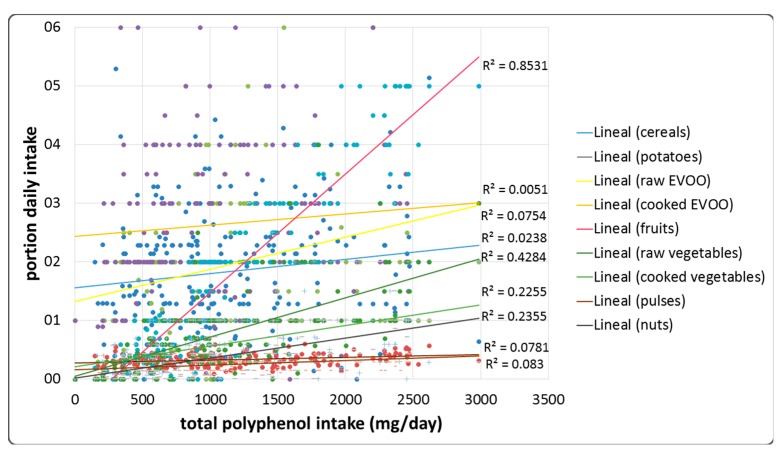
The relationship between different food groups (expressed as portion per day) and the estimated total polyphenol intake (mg/day). *R*^2^: coefficient of determination.

The main dietary sources of total polyphenols were fruits (49%) and vegetables (30%). Nuts and pulses were the third most important contributors of total polyphenol intake (8% and 6% respectively), whereas cereals, potatoes and EVOO accounted for a lower percentage of the total amount of polyphenols in the diet of the study population (Figure 2). The relationship between the different food groups and the total dietary polyphenol intake is illustrated in Figure 3.

### 3.3. Relationship between Total Polyphenol Intake, Urine 8-Isoprostanes, and Antioxidant Capacity

**Figure 4:**
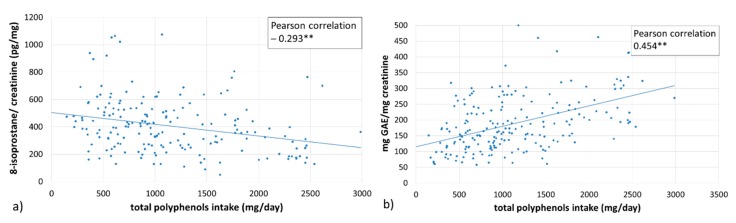
The correlation between estimated total polyphenol intake (mg/day) and; (**a**) the levels of 8-isoprostanes in urine (pg/mg creatinine), (**b**) the total antioxidant capacity expressed as mg gallic acid equivalents (GAE)/mg creatinine in the urine of the participants. ** *p* < 0.01.

Figure 4 shows the correlation between total polyphenol intake (estimated from the consumption of different Mediterranean foods and the data of Phenol Explored Data Base), the levels of 8-isoprostanes (pg/mg creatinine) in the urine of participants, and the total urine antioxidant capacity (mg GAE/mg creatinine). As 8-isoprostane is a biomarker of lipid peroxidation, it is useful to evaluate oxidative stress. In our data, dietary effects on urinary 8-isoprostane correlated significantly with the total polyphenol intake (correlation coefficient *r* = − 0.2927; *p* < 0.001) and GAE levels in urine (correlation coefficient *r* = 0.4541; *p* < 0.001). However, no significant correlations were established between urine 8-isoprostanes and the levels of physical activity of the subjects.

### 3.4. Relation between the Intake of Food Groups, Urine 8-Isoprostanes, and Antioxidant Capacity

Table 1 shows the correlatons between the different food groups and the AMDI with the urine 8-isoprostanes and the levels of GAE in urine.

**Figure 5:**
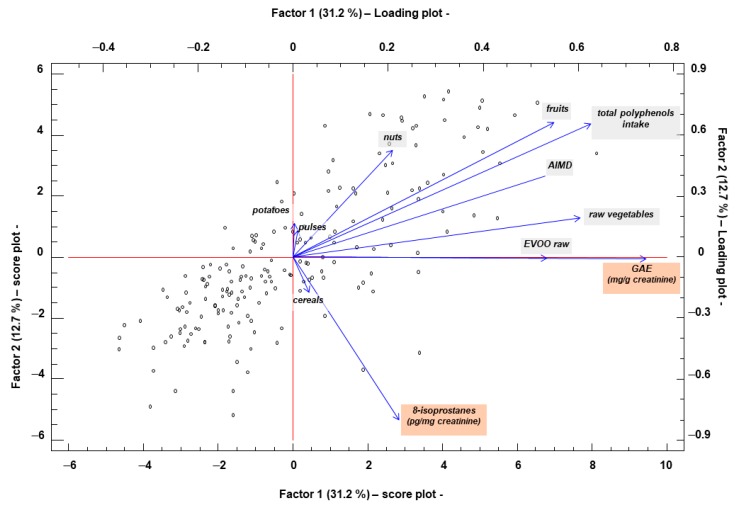
Factorial analysis biplot (score plot + loading plot) Factor 1 vs. Factor 2. Factor 1 (31.2% variance) is represented by the variables: Gallic acid equivalent (GAE, mg/mg creatinine, load = 0.745), total polyphenols intake (mg/day, load = 0.627), adherence to Mediterranean diet index (AMDI, load = 0.530), raw extra virgin olive oil (raw EVOO, portions/day, load = 0.535), fruits (portions/day, load = 0.550), and raw vegetables (portions/day, load = 0.606). Factor 2 (12.7% variance) is represented by urine 8-isoprostanes (pg/mg creatinine, load = −0.803), total intake of polyphenols (mg/day, load = 0.654), fruits (portions/day, load = 0.664), and nuts (portions/day, load = 0.526).

To perform the Factorial Analysis, the variables that could be related to the oxidative processes (GAE, 8-isoprostanes and total polyphenol intake) were selected together with the AMDI and the ingested rations/day of the different raw foods (EVOO, fruits, vegetables, legumes, nuts, cereals, and potatoes). Four factors were obtained that explain 64% of the variability of the data. Factor 1 (31.2% variance) is represented by the loads of the variables GAE (0.745), total polyphenols intake (0.627), AMDI (0.530), EVOO raw (0.535) fruits (0.550) and raw vegetables (0.606). For the value of the loads of these variables, Factor 1 could be defined as “antioxidant capacity”. Factor 2 (12.7% variance) is represented by 8-isoprostanes (−0.803), intake of total polyphenols (0.654), fruits (0.664) and nuts (0.526). For the value of the loads of these variables, Factor 2 could be defined as “evaluation of oxidative stress”. The representation of score plot with respect to Factor 1 and Factor 2 and the loading plot is shown in Figure 5.

## 4. Discussion

The traditional Mediterranean diet includes mainly plant foods and EVOO as the more important sources of phytosterols, which play a pivotal role in slowing down oxidative stress, and contribute to the prevention of chronic diseases, in particular cardiovascular diseases, cancer and metabolic syndrome [23,24,25]. The results obtained from our study population show a daily intake of bread, pasta, rice, vegetables, fruits, pulses, and nuts, that is lower than recommended [26]. These results are in accordance with the mean value of adherence to the Mediterranean diet (8.6, 9.2 and 9.8, respectively, for the age groups, in a scale from 0 to 14), and align with the results of other studies [27,28]. The main deviations from Mediterranean dietary patterns were the low intake of vegetables and fruits, and the high consumption of meat, dairy products, soft drinks, and pre-cooked food. Only EVOO daily intake was above recommended levels.

According to these results and the data shown in the Phenol Explored Data Base, the mean of total polyphenol intake (Folin method) was 1098 ± 45 mg/day. Main food contributors were fruits (49%), vegetables (30%), nuts (8%), pulses (6%), cereals (3%), and EVOO (1.5%), in accordance with previous studies [29,30]. The high dietary intake of polyphenols has been inversely associated with cardiovascular disease, cancer, metabolic diseases, and mood disorders [3,4,11,12,31,32]. The most investigated molecular mechanisms of polyphenols are in relation to their antioxidant activity. Several studies have established the molecular mechanisms that explain the protective effects of polyphenols, attributing their antioxidant capacity to the regulation of redox enzymes and the reduction of reactive oxygen species (ROS). These effects have been related to the intake of food groups that are included in Mediterranean diet. A high dietary intake of whole grain food improves postprandial glucose and insulin homeostasis [33], and EVOO (which is high in polyphenols levels) has a cardioprotective effect [34,35,36]. Indeed, we established a significant and positive regression between total polyphenols in the diet and the daily intake of fruits, vegetables (raw and cooked), pulses, and nuts. Daily intake of EVOO did not correlate with total polyphenols in the diet, but a positive relationship was established when we only considered raw EVOO.

The measurement of total antioxidant capacity in urine using the Folin-Ciocalteu method has been validated as a way to estimate levels of polyphenol consumption, which has in turn been related to several pathologies, such as obesity and hypertension [15,22,37]. Both the measurements obtained from collecting 24-h urine or spot urine normalized by creatinine, have been used to estimate total polyphenol intake [20]. Our results support this method as a biomarker of total dietary polyphenols, based on the positive and significant regression that was established between estimated polyphenol intake and the mg of GAE/mg of creatinine in the urine samples of participants.

In addition, a significant but negative lineal regression was established between total dietary polyphenols and the levels of urinary 8-isoprostanes. The 8-isoprostanes in urine are used as a biomarker of oxidative status, specifically for lipid oxidative damage [38], and its decrease has been associated with the consumption of foods rich in polyphenols, such as EVOO [15].

Total antioxidant capacity in urine correlates positively with the daily intake of fruits, vegetables, nuts, raw EVOO, and with adherence to the Mediterranean diet. However, levels of urinary 8-isoprostanes correlate negatively with the daily intake of fruits, nuts, and with the AMDI.

Finally, the factor analysis showed that the urinary indexes (GAE mg/mg creatinine and 8-isoprostanes pg/mg creatinine) used in the present study could be used as markers of both total dietary polyphenol intake, and the consumption of foods typical of the Mediterranean diet, such as EVOO, fruits, and raw vegetables.

However, our study has some limitations: We enrolled exclusively healthy women from a specific area (South Spain), the current experimental design (cross-sectional study) regarded only the associations between variables, and other urinary markers could have been included in the analysis. Future studies in this field could increase sample sizes, analyze other populations (e.g., compare males and females, inside and outside the Mediterranean area), analyze other lifestyle factors, evaluate different dietary markers in urine samples, and perform interventional studies with various dietary patterns to allow us to establish cause-effect relationships.

## 5. Conclusions

Taken together, these results demonstrate that adherence to the Mediterranean diet and the high intake of typical Mediterranean food groups with elevated polyphenol content, are associated with antioxidant status and improved markers of lipid oxidative stress in healthy women. In addition, the results indicate that the measurement of total antioxidant capacity in urine, and 8-isoprostanes levels in urine may be suitable biomarkers of total polyphenol intake.

## Figures and Tables

**Table 1:** The correlations between different food groups and the AMDI, and the urine 8-isoprostane and the levels of gallic acid equivalents (GAE) in urine.

P	Cereals	Potatoes	EVOO (Raw)	EVOO (Cooked)	Fruits	Vegetables (Raw)	Vegetables (Cooked)	Pulses	Nuts	AMDI
8-isop/creat (pg/mg)	0.0905	−0.0174	−0.0680	0.1249	−0.3079 ***	−0.0313	−0.1834 *	−0.0579	−0.1997 **	−0.2070 **
GAE/creat (mg/mg)	0.1061	0.1116	0.2379 ***	0.0860	0.4183 ***	0.2777 ***	0.2143 **	0.0871	0.2365 ***	0.4064 ***

Pearson Correlations between the levels of urine 8-isoprostanes (expressed as pg/mg creatinine) and different food groups (expressed as portions per day) and the adherence to the Mediterranean Diet (AMDI); and the levels of urinary equivalents of gallic acid (GAE) (expressed as mg/mg creatinine) and different food groups (expressed as portions by day) and the adherence to the Mediterranean Diet. * *p* < 0.05, ** *p* < 0.01, *** *p* < 0.001.
